# Prediction of Conductivity by Adaptive Neuro-Fuzzy Model

**DOI:** 10.1371/journal.pone.0092241

**Published:** 2014-03-21

**Authors:** S. Akbarzadeh, A. K. Arof, S. Ramesh, M. H. Khanmirzaei, R. M. Nor

**Affiliations:** 1 Centre for Ionics University Malaya, Department of Physics, University of Malaya, Kuala Lumpur, Malaysia; 2 Department of Physics, Faculty of Science, University of Malaya, Kuala Lumpur, Malaysia; National Research Council of Italy, Italy

## Abstract

Electrochemical impedance spectroscopy (EIS) is a key method for the characterizing the ionic and electronic conductivity of materials. One of the requirements of this technique is a model to forecast conductivity in preliminary experiments. The aim of this paper is to examine the prediction of conductivity by neuro-fuzzy inference with basic experimental factors such as temperature, frequency, thickness of the film and weight percentage of salt. In order to provide the optimal sets of fuzzy logic rule bases, the grid partition fuzzy inference method was applied. The validation of the model was tested by four random data sets. To evaluate the validity of the model, eleven statistical features were examined. Statistical analysis of the results clearly shows that modeling with an adaptive neuro-fuzzy is powerful enough for the prediction of conductivity.

## Introduction

Electrochemical impedance spectroscopy (EIS) is at the heart of our understanding of electrochemical characteristics of materials. It determines diffusion impedance, rate of charge transfer, charge transport processes, double-layer capacitance and solution resistance [1]–[4]. One of the important factors which can be obtained by EIS is conductivity. Bulk and interface physical properties, such as mechanical, compositional, and crystallographic properties, and specifically, electrical–change impulsive and heterogeneous distributions of charge (polarizations), decrease the overall conductivity of a system [5]–[9]. Owning to non-linear relationships involved in the calculation of conductivity, most of the regression models are not applicable in the prediction of conductivity.

Fuzzy logic is a powerful tool that can be used to model complex systems [10], [11]. It has become popular in the modeling of different physical systems, including solar cells, fuel cells, supercapacitors, corrosions and batteries [12]–[16]. However, far less attention has been paid to employing this method in impedance spectroscopy, despite combination of these two methods providing a knowledge base which helps to save time, energy and material. Fuzzy logic normally follows five steps. Firstly, input membership functions (MFs) are assigned to input characteristics. Secondly, the membership functions are linked to rules. Thirdly, the rules are connected to output characteristics and then output membership functions are assigned to the output characteristics. Finally, a one-valued output is concluded from the output membership functions [11], [17].

Determining the membership functions and assigning rules is sensitive work which requires expert knowledge. Artificial neural networks can facilitate the automatic determination of membership functions and rules. The combined structure is called a neuro-fuzzy inference system (ANFIS) [18], [19]. ANFIS provides a method for fuzzy logic modeling to obtain information about input/output data sets [20]. Accordingly, in cases where the results show acceptable statistical features, the model can be applied to desired sets of inputs to predict their outputs.

In this study conductivity was modeled in terms of four inputs. Focus in the field of electrochemistry has changed from a time and concentration interdependence, to frequency domain [2], thus, one of the input factors chosen was frequency. As electrical response can differ significantly depending on the nature, texture and microstructure of polymer films, and the types of charge present [21], two other input factors selected were the thickness and weight percentage of salt in the polymer films. Although a considerable amount of literature deals with measurements made at room temperature [22]–[25], the temperature is effectively a parameter in EIS and is therefore considered as the last input. These four inputs are used to predict the ac-conductivity of a polymer film; a development toward small-signal ac analyses.

The statistical characteristics examined included mean-absolute error (MAE), mean-square error (MSE), root mean-square error (RMSE), the factor of two (FA2), percentile variance (VAF), index of agreement (IA), systematic and unsystematic MSE (MSES and MSEU, correspondingly), intercept and slope and determination coefficient (R^2^) and intercept and slope of linear regression between model and experimental data [26].

## Methodology

### Experimental Data Collection

Rice starch (Sigma-Aldrich) as a biopolymer and lithium iodide salt (Aldrich, crystalline powder, 99.9% trace metal basis) were used in this work. Each sample was prepared using a solution cast technique. Rice starch (1 g) was dissolved in 25 ml of DI-water and stirred and heated to 80°C until gelatinization. After gelatinization, the solution was cooled to room temperature with continued stirring and different amounts of lithium salt (LiI) were added (5, 10, 15, 20, 25, 30 and 35 wt. %). The solution was stirred to obtain a homogenous mixture. Solutions were then cast onto Teflon petri dishes and oven dried at 60°C for 24 h. After drying, solid films were cast.

Conductivity and frequency-dependent studies were carried out using an electrochemical impedance spectroscopy (EIS) analyzer (Hioki, 3532-50 LCR HiTESTER) with a frequency range of 50 Hz to 5 MHz. The samples were compressed between two stainless steel blocking electrodes with areas of 2.98 cm^2^. The imaginary parts of impedance were automatically computed with a LCR HiTESTER. The ionic parameter of ac-conductivity was calculated using following equation.
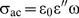
(1)where 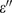
is the imaginary part of impedance known as dielectric loss, ω is angular frequency and 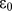 is the permittivity of the free space [27].

### Modeling Design

**Figure 1:**
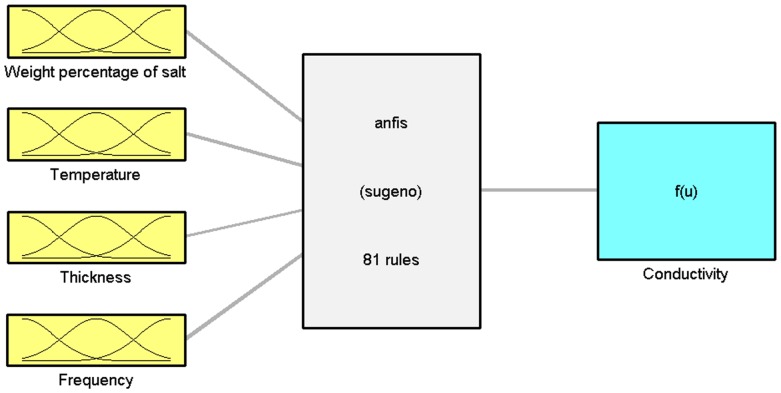
General neuro-fuzzy arrangement for conductivity modeling.

Artificial neural network techniques have been extensively applied in different fields of study [28]–[30]. Nevertheless, one of the major shortcomings in the application of these techniques is lack of interpretation [31]. Fuzzy logic is another method for modeling which can cover interpretation weakness in neural networks. The fuzzy inference system is comprised of premises and antecedents, which are connected by fuzzy rules. The structure of fuzzy logic is in a sense analogous to neural network modeling. It represents inputs through input membership functions and their related parameters, then maps output membership and related parameters of the output membership functions. This system can be applied to interpret the input and output maps. The commercial Fuzzy Logic Toolbox within the framework of MATLAB version of 7.12.0.635 was used throughout the study. The extra code is written to get the optimum desire results from the software. Figure 1. Shows the schematic structure used for modeling with neuro-fuzzy in the present study.

Sugeno is a form of fuzzy inference that the consequence of each rule consequent is a constant or linear combination of the inputs. The output variables are gained by using fuzzy rules to the fuzzy input sets. Since it is supposed to use ANFIS, only zero-order or first-order Sugeno fuzzy inference system can be applied. The samples of zero-order and first-order rules are as follows [32]–[34]:
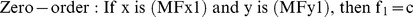
(2)




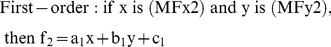
(3)


**Figure 2:**
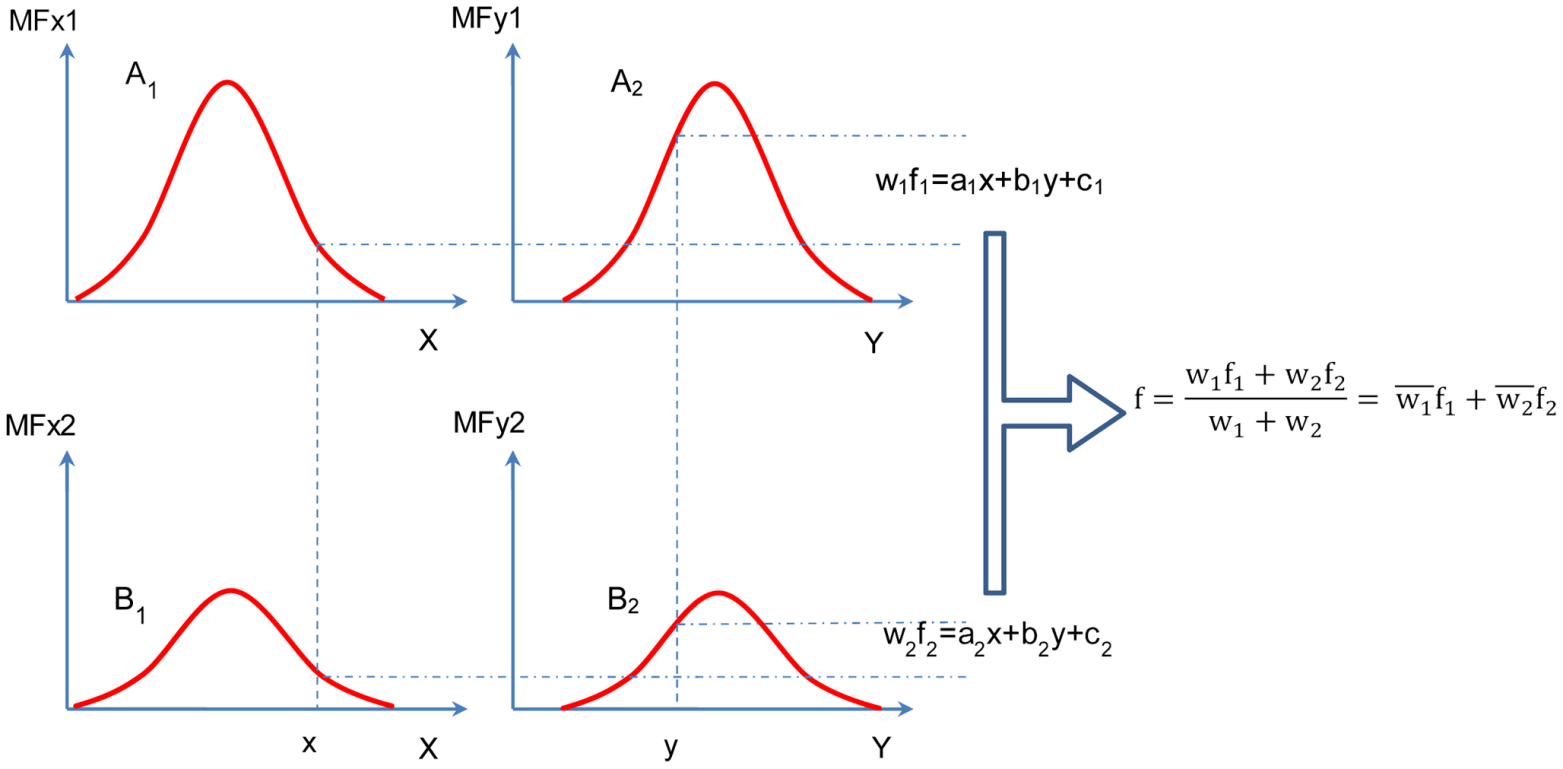
First-order Sugeno model with two inputs and two rules.

**Figure 3:**
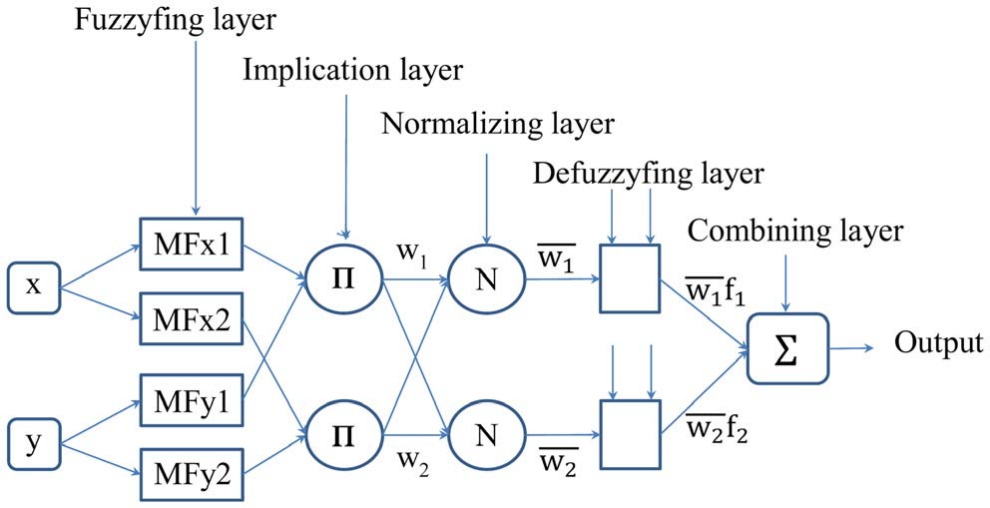
Schematic ANFIS structure for two inputs and two rules.

In order to calculate the output, the weighted linear mixture of the consequent is computed. A schematic combining the first-order Sugeno model with two inputs and two rules is shown in figure 2 [35] and the corresponding ANFIS structure is depicted in figure 3 [32].

The first layer is the input layer. In the schematic shown figure 3 the input layer consists of two inputs, x and y, however, in actual modeling, the input layer embraces four factors, namely: frequency, film thickness, weight percentage of salt in the polymer, and temperature.

The second layer is the fuzzyfing layer which allocates membership functions to inputs. In fuzzy theory, membership functions are linguistics labels which transform verbal data to numeric data, whereas in the present study membership functions are just part of the modeling. Three Gaussian membership functions were used for each input as follows:
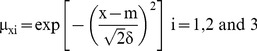
(4)where m and δ show the mean and the variance of Gaussian function and i indicates the number of membership function related to input x. Parameters in the fuzzyfing layer are denoted as the premise parameters and are nonlinear parameters.

The third layer is the implification layer. Two methods can apply to this layer; minimum and product methods, but the product method is most commonly applied. Here, the incoming inputs from the previous layer are multiplied and applied to an antecedent formula to produce the outputs. This process can be shown by the following formula:
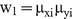
(5)


μ_x_ and μ_y_ are membership functions of input 1 and 2. In this modeling study, the four membership functions multiply together.

The fourth layer is a normalizing layer. In order to normalize the output value, the implication parameter, w, should be normalized. The normalization follows the formula below.
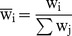
(6)


For the schematic, input x and y simplify to 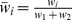 in which i = 1,2. The 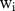 calculates the proportion of the ith rule’s strength to the total summation of all rules’ strengths.

The next layer is a defuzzyfing layer. It applies 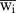 to linear function *f*. For two inputs, the definition of *f* is based on a, b and c antecedent linear parameters. When n input parameters exist, there are n+1 antecedent parameters.
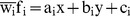
(7)


The backpropagation neural network adjusts in order to obtain the desired output which matches to the target experimental data.

The last layer is the total summation of 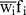, which provides the final output [34].

The output shape varies with premise and antecedent parameters. Neuro-adaptive learning helps to automatically adjust these parameters by applying a backpropagation algorithm alone or with a least squares method (hybrid method). This allows fuzzy systems to learn from the data they are modeling. The membership functions parameters will change during the learning process. A gradient vector enables the tuning of these parameters. This vector presents a measure to evaluate the quality of the fuzzy system in the modeling of the given input and output sets. Once the gradient vector was found, any of the standard optimization methods could be used to tune the parameters in order to decrease error. Generally, the sum of the squared differences between experimental and model outputs is used for error measurement. In this paper, the conductivity estimation was performed using a hybrid learning algorithm.

Logarithmic pretreatment was applied to the raw frequency data to convert them from high range to normal range. The data sets were separated randomly into three subsets; training, checking and testing data sets. Overfitting contributes to high testing error and can lead to poor prediction. Two solutions can be considered to avoid overfitting. Firstly is the use of a large random number of input data for the training set. In this paper, the total number of random training sets was 6830. The second solution is to use data which check the error measurement of checking and training data after each epoch. Here, 3400 checking data were randomly chosen for training purposes. The model performance was tested via separate test data sets randomly chosen from the experimental data set. 3400 data were selected for the testing set.

## Results and Discussion

**Figure 4:**
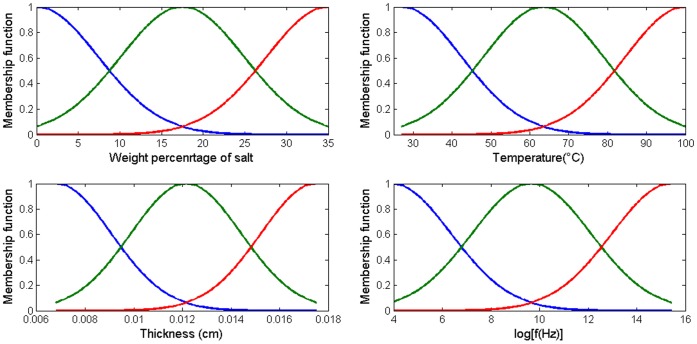
The optimum membership functions for frequency, salt weight percentage, thickness and temperature in three levels.

Three Gaussian membership functions were chosen for each input. After optimizing with a hybrid learning algorithm, the parameters of the optimum membership functions were obtained. figure 4 shows the optimal Gaussian function in terms of the lowest mean squared error in three levels; low, medium and high.

In the present study the statistical features were employed to measure the goodness of the model. In the literature, different descriptive statistical values have been used for model evaluation. These features include MAE, MSE, RMSE, FA2, VAF, IA, MSES and MSEU, intercept and slope and R^2^ of linear regression between model and experimental data [26], [36]–[38].

MAE is a measurement used to determine how close the model data are to the experimental data and is defined as:
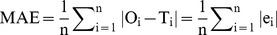
(8)


As the name implies, the mean absolute error is an average of the absolute errors 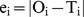, where 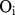 is the model output, 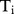 the experimental target value, and n the number of data.

MSE is another way to measure the difference between model values and the experimental values. It is defined as an average of the squared errors using the following formula:
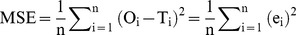
(9)


RMSE, also distinguished as the quadratic mean of errors, is another statistical gauge of the amount of error.
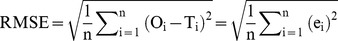
(10)


FA2 is expressed as a fraction of the data in which the ratio of model to experimental data is between 0.5 to 2%.
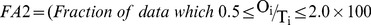
(11)


The ideal value for this statistical feature is 100 percent.

Another factor to measure the correctness of a model is the percentile variance. The percentile variance (VAF) computes the variance accounted for among two sets of data. The VAF between experimental target data *T_i_* and the model data *O_i_* is expressed as.
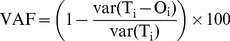
(12)where var indicates the variance among the data.

IA is a standardized measurement of the model prediction error. It varies from zero to one. It can be calculated from the following formula:
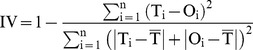
(13)where 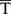 is the mean of the measured values. A value of one reveals a perfect agreement between the experimental and predicted values, while 0 shows no agreement at all [38]. The index of agreement can find additive and proportional alterations in the measured and modeled means and variances; although, IV is overly sensitive to extreme quantities owing to the squaring of the differences.

When a linear regression exists between model and experimental values, the linear regression line pass best fit from them. These lines can be different to the both measured and model data. These errors are measured using MSES and MSEU, defined as:
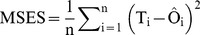
(14)




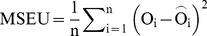
(15)Where
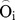
is the predicted value obtained from the linear regression 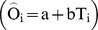


Table 1 indicates the above statistical features for the current modeling.

**Table 1:** The summation of statistical characteristics to evaluate the neuro-fuzzy modeling with experimental data.

	MAE	MSE	RMSE	FA2	VAF	IA	MSES	MSEU
Train data	1.72E-06	1.48E-11	3.84E-06	83.62	99.88	0.99970	2.50E-25	1.48E-11
Check data	1.86E-06	2.28E-11	4.77E-06	83.08	99.82	0.99955	1.19E-13	2.30E-11
Test data	1.75E-06	1.80E-11	4.24E-06	83.07	99.86	0.99964	1.49E-15	1.80E-11
Total data	1.76E-06	1.76E-11	4.19E-06	83.35	99.86	0.99965	6.18E-15	1.76E-11

The average results are 4.98E-05, 5.03E-05, 4.92E-05 and 4.98E-05 for train, check, test and total data, respectively. By comparing the error measurement with average data, it can be seen that MAE and RMSE are one order less than the average data and MSE is six orders less. This result is acceptable. From the factor of two index, it can be seen that 83% of predicted data is neither lower than half nor greater than twice the real data. From the percentile variance, it can be seen that the prediction error variance is almost the same as the measured error variance, with almost 100% accuracy. The index of agreement shows that error in the model data with 99.99% accuracy is the same as error in the real data. The MSES and MSEU imply the amount that the experimental and modeling data differ from the normal trend. The sum of these two errors gives the mean squared error.

**Figure 5:**
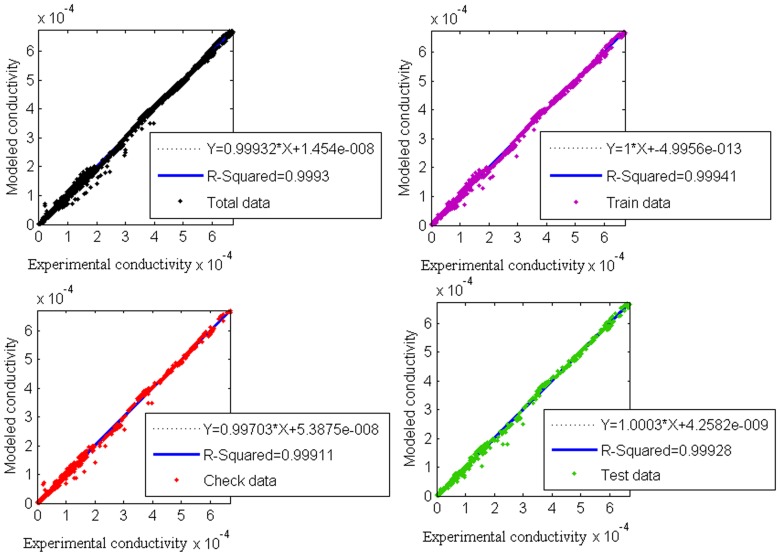
Regression plots of model and experimental data for four datasets: Total, Train, Check and Test values.

The slope of the linear regression of the parameters, the y-intercept and R^2^ are used to assess the correctness of a model. The y-intercept and slope of the linear regression line can show how well model data match experimental data. The slope shows the relative association between model predictions and measured data and experimental values. Since the perfect model is the one in which they are same, the ideal slope is one. The y-intercept shows the existence of a lag between the model and experimental values; a value of zero implies that the data are not completely aligned. Pearson’s determination coefficient R^2^ describes the degree of linearity between model and experimental data. The determination coefficient, which ranges between zero and one, is an index of the relationship between observed and model and normally values greater than 0.5 are considered acceptable. However, when this value is close to one a strong relationship between the model and measured data is implied, and this links to the power of the model. Figure 5 shows that the slope is very close to a value of one and the intercept is four orders less than the average data. Moreover, the determination coefficient suggests a strong relationship between the model and experimental data. By comparing these results for train, check, test and total data sets, it can be understood that the model is not only able to predict the current experimental results but also can be applied for unknown future data.

**Figure 6:**
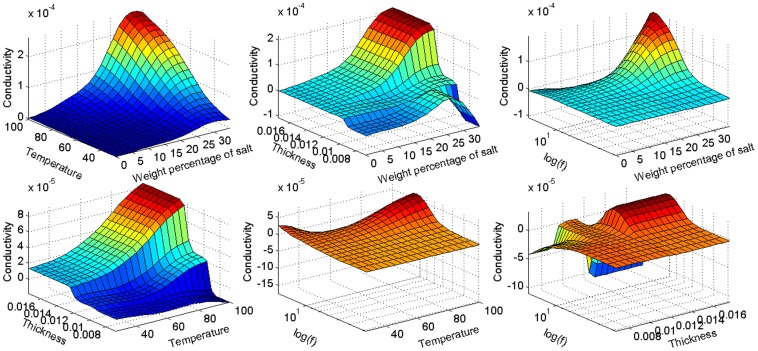
Model prediction of changing conductivity in terms of four experimental factors; weight percentage of salt, temperature, thickness of film and logarithm of frequency.

Figure 6 illustrates the results obtained from the modeling of conductivity. The figure depicts the trend of variation in conductivity based on experimental factors. The three factors of weight percentage of salt, temperature and thickness are shown with a normal scale, while the frequency parameter is shown with a logarithmic scale. This figure provides information about the surface trend and optimum point of the experiment. Furthermore, it shows the possible error in preliminary experiments. As can be seen in the graphs, surface plot of the thickness fluctuates, while the other parameters have a smooth surface. It is likely that there were some errors in the measurement of thickness due to equipment inaccuracy or human error. These plots also provide information about the importance of parameters. Parameters that exhibit a steep slope have more significance since a slight change in the parameter causes large variation in results.

## Conclusions

This paper presents a neuro-fuzzy model to predict conductivity of polymer film. The key to this model is to forecast conductivity in terms of basic experimental factors, namely frequency, temperature, thickness of the polymer film and weight percentage of salt. The model is validated with three sets of data; training, checking, testing data set. The performance of model evaluated with several statistical characteristics. These characteristics included error measurement, variance measurement, model evaluation and linear regression between model results in four sets of data; train, check, test and total dataset and the experimental data. All the statistical features imply the correctness and power of the model. The model can be applied as an advisory system to inform researchers about trends and possible optimum points, error measurement and the importance of factors.
